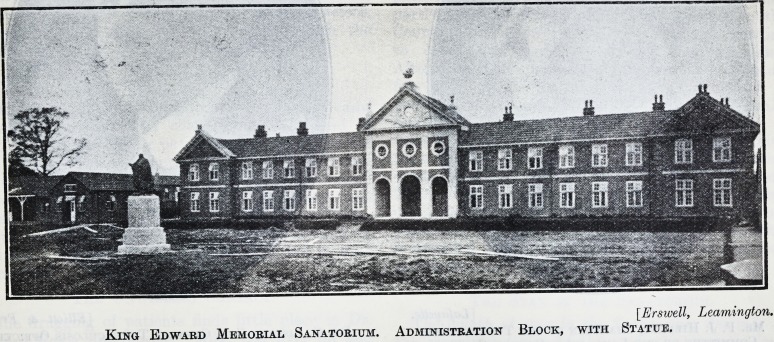# Warwickshire's Memorial to King Edward VII

**Published:** 1924-08

**Authors:** 


### WARWICKSHIRE'S MEMORIAL TO KING EDWARD VII.

The Warwickshire King Edward VII. Sanatorium for the treatment of tuberculosis, which was opened by the Duke of York recently, is a fine building situated at Hertford Hill, about three miles distant from the county town, of which it commands a good view. It was finished last October and is now fully occupied, 203 patients being cared for. The work of construction was carried out under the auspices of a joint committee, and the total cost (including the price of the land) has been nearly £100,000. Financial help has been given by the Red Cross Society, and a gift of nearly £30,000 came from a private benefactor, Mr. John Alfred Watson, of Chadwick Manor, Knowle. The late King was keenly interested in the work of sanatoria for the treatment of consumption, and it was as a permanent and useful memorial to him that the institution was built.

[*Erswell, Leamington.*

King Edward Memorial Sanatorium. Administration Block, with Statue.

### A Credit to the County.

The Duke congratulated all concerned on the splendid example set in "providing this valuable and necessary institution for the care of the sick and suffering. This wonderful building speaks well for the high ideals and public-spirited sentiments of the people of this county." His Royal Highness, in addition to opening the sanatorium, unveiled a statue of King Edward VII. and a bust of the late Lord Hertford, who initiated the scheme.

## Figures and Tables

**Figure f1:**